# *R-BPMV*-Mediated Resistance to *Bean pod mottle virus* in *Phaseolus vulgaris* L. Is Heat-Stable but Elevated Temperatures Boost Viral Infection in Susceptible Genotypes

**DOI:** 10.3390/v13071239

**Published:** 2021-06-26

**Authors:** Chouaïb Meziadi, Julie Lintz, Masoud Naderpour, Charlotte Gautier, Sophie Blanchet, Alicia Noly, Ariane Gratias-Weill, Valérie Geffroy, Stéphanie Pflieger

**Affiliations:** 1 Université Paris-Saclay, CNRS, INRAE, Univ Evry, Institute of Plant Sciences Paris-Saclay (IPS2), F-91405 Orsay, France; chouaib.meziadi@corebiogenesis.com (C.M.); julie.lintz@univ-lorraine.fr (J.L.); m.naderpour@areeo.ac.ir (M.N.); charlotte-gautier60@hotmail.fr (C.G.); sophie.blanchet@universite-paris-saclay.fr (S.B.); alicia.noly@universite-paris-saclay.fr (A.N.); ariane.gratias-weill@universite-paris-saclay.fr (A.G.-W.); valerie.geffroy@universite-paris-saclay.fr (V.G.); 2 Université de Paris, CNRS, INRAE, Institute of Plant Sciences Paris-Saclay (IPS2), F-91405 Orsay, France; 3 Seed and Plant Certification and Registration Research Institute, Agricultural Research, Education and Extension Organization, Ministry of Agriculture, Allameh Jafari Blvd, Karaj, Iran

**Keywords:** *Bean pod mottle virus*, *Phaseolus vulgaris* L., heat-stable resistance, high temperature

## Abstract

In the context of climate change, elevated temperature is a major concern due to the impact on plant–pathogen interactions. Although atmospheric temperature is predicted to increase in the next century, heat waves during summer seasons have already become a current problem. Elevated temperatures strongly influence plant–virus interactions, the most drastic effect being a breakdown of plant viral resistance conferred by some major resistance genes. In this work, we focused on the *R-BPMV* gene, a major resistance gene against *Bean pod mottle virus* in *Phaseolus vulgaris*. We inoculated different BPMV constructs in order to study the behavior of the *R-BPMV*-mediated resistance at normal (20 °C) and elevated temperatures (constant 25, 30, and 35 °C). Our results show that *R-BPMV* mediates a temperature-dependent phenotype of resistance from hypersensitive reaction at 20 °C to chlorotic lesions at 35 °C in the resistant genotype BAT93. BPMV is detected in inoculated leaves but not in systemic ones, suggesting that the resistance remains heat-stable up to 35 °C. *R-BPMV* segregates as an incompletely dominant gene in an F2 population. We also investigated the impact of elevated temperature on BPMV infection in susceptible genotypes, and our results reveal that elevated temperatures boost BPMV infection both locally and systemically in susceptible genotypes.

## 1. Introduction

Among all plant diseases, viruses account for about half of all known pathogens [1] and constitute particular entities, non-living organisms that are obligate and intra-cellular parasites that need a live host for replication. Plant viruses are biotic pathogens that cause serious epidemics in major crops with annual yield losses of more than $30 billion [2]. No effective pesticide-based control measures are effective against viruses, so the most reliable method of plant protection is increasing plant genetic resistance [2]. Plant resistance relies on different immune responses. First, recognition of viral double-stranded RNAs is a major mechanism in antiviral plant defense [3] that induces both RNA silencing [4] and pattern-triggered immunity [5,6,7]. Viruses have evolved viral suppressors of these responses in order to promote their own replication [8,9,10,11]. Second, plant effector-triggered immunity (ETI) is induced by the recognition of virus effectors by resistance (*R*) proteins mostly represented by nucleotide-binding domain leucin-rich repeat containing receptors (NLRs) [12]. NLR receptors are intracellular and encoded by dominant genes [13]. Many *R* genes against viruses have been identified so far, as well as the corresponding viral-encoded effectors that are subsequently referred to as avirulence (Avr) factors [14,15]. Other types of resistances are also effective against viruses such as those controlled by quantitative trait loci [16] or ‘recessive genes’, the latter ones encoding mutated or truncated host factors that are hijacked by viruses in their wild type forms for their life cycle [17].

Plant resistance is strongly affected by environmental conditions, and temperature is undoubtedly one of the key parameters that have a major impact on worldwide plant production. It is well known that elevated temperatures influence plant–virus interactions, as well as the timing and severity of disease epidemics [18,19,20], but little is known about the associated molecular mechanisms in plant–pathogen interactions [21]. The more drastic impact of elevated temperatures is the breakdown of plant viral resistance conferred by major *R* genes [18]. Indeed, some *R* genes have been shown to be overcome at high temperatures, usually around 28–30 °C, allowing viral infection and spreading at the whole-plant level. This is the case for the *N*-mediated resistance that occurs only at temperatures below 28 °C in *Nicotiana tabacum* [22] and for the *Tsw*-gene-mediated resistance that is overcome between 25 and 30 °C in *Capsicum* species [23]. Conversely, for other *R* genes, resistance is still efficient at temperatures above 28–30 °C, such as, for example, the *Rx*-mediated resistance that is not compromised against *Potato virus X* (PVX, *Potexvirus*) at temperatures up to 32 °C [24]. Alternatively, elevated temperatures may also induce more mild effects on plant resistance conferred by *R* genes. Indeed, the resistance phenotype can be modified, meaning that local necrotic lesions such as hypersensitive reaction (HR, a form of programmed cell death [25]) can be transformed into systemic necrosis. Additionally, extreme resistance, which inhibits virus replication without apparent HR, can be shifted to HR or even to systemic necrosis at temperatures above 28–30 °C. In the absence of *R* genes, elevated temperature was shown to promote symptom severity, systemic spreading, and replication potential of viruses [26,27,28]. Other studies reported contradictory results in the sense that elevated temperatures reduced viral symptoms and viral accumulation [29,30,31], which was hypothesized to be attributable to increased efficiency of the RNA silencing pathway at elevated temperature [32]. Therefore, predicting a general outcome for all the pathosystems may be difficult.

Common bean (*Phaseolus vulgaris* L.) is a major pulse crop of agronomic importance cultivated as a dry grain or fresh vegetable. Indeed, it is the most important grain legume for human consumption worldwide, especially in developing countries such as Central and South America and Southeastern Africa [33]. Common bean varieties are grown over a wide range of latitudes but optimal growth conditions need temperatures comprised between 17.5 and 23 °C [34,35]. Indeed, daytime temperatures above 30 °C and nighttime temperatures above 22 °C lead to yield losses [36,37].

In the main production areas of common bean, several viruses including *Potyviruses Bean common mosaic virus* (BCMV), *Bean common mosaic necrosis virus* (BCMNV), *Bean yellow mosaic virus* (BYMV), and *Clover yellow vein virus* (ClYVV) affect the quality and quantity of bean productions (reviewed in [38]). The well-known *I* locus, located at the extremity of chromosome 2, confers resistance to a large part of them. In addition to resistance against *Potyviruses*, resistance to *Comovirus* has also been positioned in the region of the *I* locus [39,40]. This is the case of the *R-BPMV* gene conferring resistance to *Bean pod mottle virus* (BPMV) in *P. vulgaris* genotype BAT93 [41].

The aim of the current study was to address several questions concerning the *R-BPMV*-mediated resistance in *P. vulgaris*. Does the *R-BPMV*-mediated resistance to BPMV depend on temperature? If so, at which temperature does the phenotype switch occur? What is the inheritance of the *R-BPMV* gene? Here, we report that *R-BPMV* induces HR lesions at 20 °C. Further analysis revealed that at 25 and 30 °C, *R-BPMV*-mediated resistance still induces HR lesions, whereas at 35 °C, local HR lesions are replaced by chlorotic lesions but resistance is not overcome at the whole-plant level. Finally, we also investigated the impact of elevated temperature on BPMV infection in a compatible context (i.e., in susceptible genotypes), and our results highlighted that rising temperature boosts BPMV infection.

## 2. Materials and Methods

### 2.1. Common Bean Material and Growing Conditions

The following genotypes of *Phaseolus vulgaris* were used in this study: BAT93 (Mesoamerican breeding line), JaloEEP558, Black Valentine (both Andean landraces), and two Near-Isogenic Lines for the *I* locus [42], Black Turtle 1 (BT-1; *I/I*, resistant to BCMV and BCMNV) and Black Turtle 2 (BT-2; *i/i* susceptible to BCMV and BCMNV). The inheritance of *R-BPMV* was studied using 60 F2 individuals derived from a cross between BAT93 (*R-BPMV/R-BPMV*, resistant to BPMV) and the Andean landrace JaloEEP558 (*r-bpmv/r-bpmv*, susceptible to BPMV).

Growing conditions from sowing to virus inoculation were followed as described in Pflieger et al. [41] with some modifications. Briefly, seeds were sown in soil instead of vermiculite and grown in a growth chamber at 23 °C under a 16 h light/8 h dark cycle and 75% relative humidity until the BPMV-inoculation stage (fully expanded primary leaf stage, 10 days post-sowing in our growth conditions). From sowing to inoculation, seedlings were watered with tap water.

### 2.2. Viral Material

An infectious BPMV cDNA clone derived from isolate IA-Di1 was provided by C. Zhang and S. Whitham (Iowa State University, Ames, IA, USA) and described previously [43]. Briefly, the infectious clone BPMV-WT contains the WT-RNA1 (on infectious plasmid ‘pBPMV-IA-R1M’) and the WT-RNA2 (infectious plasmid ‘pBPMV-IA-V1’) of BPMV [43]. We also used the GFP-tagged BPMV (BPMV-GFP, infectious plasmids ‘pBPMV-IA-R1M’ + ‘pBPMV-GFP2’) [43] in which a GFP cassette was inserted in the frame between the movement protein (MP) and the large-coat protein (L-CP) coding regions and was flanked with protease recognition sites, allowing excision from the RNA2 polyprotein.

### 2.3. Viral Rub-Inoculation of P. vulgaris Plants and High-Temperature Assays

Viral rub-inoculation of *P. vulgaris* plants was performed as described previously [41,44]. Briefly, a viral inoculum was prepared by grinding frozen or fresh infected leaves from *P. vulgaris* cv. Black Valentine with a mortar and pestle in the presence of a mock buffer (potassium phosphate buffer 0.1 M, pH 7) [44]. Mechanical inoculation was then performed on one primary leaf of a healthy plant, using carborundum as an abrasive [44]. Inoculated plants were then placed in a growth chamber either at constant 20 °C (control temperature) or at constant 25, 30, or 35 °C (high temperature assays) in a growth chamber Aralab (Fitoclima 1.200, Rio de Mouro, Portugal) under 75% constant humidity at 16/8 h light/dark condition. After inoculation, plants were watered with a nutritive solution. Each experiment was reproduced at least twice.

### 2.4. Cell Death Assays

For HR assays, the leaves of 7 days post-inoculation (dpi) plants were stained with trypan blue in lactophenol solution (lactic acid:glycerol:liquid phenol:distilled water (1:1:1:1), 0.067% *w*/*v* trypan blue) in universal tubes and heated in a boiling water bath for 2 min. After cooling, the solution was replaced with chloral hydrate (2.5 g/mL), and samples were shaken until leaves were fully destained. For observations, the chloral hydrate was replaced with 60% glycerol.

### 2.5. Detection of GFP Fluorescence in Planta

GFP fluorescence in whole plants was detected by using a Black Ray long-wave UV lamp (high intensity 100-Watt long-wave UV lamp; UVP, Upland, CA, USA). Higher-magnification fluorescence detection was performed with an epifluorescence microscope for temperature assays at 20 and 25 °C (Leica MZ16F, Leica Microsystems GmBH, Wetzlar, Germany) equipped with a fluorescein isothiocyanate–tetramethyl rhodamine isothiacyanate multiband filter. The GFP fluorescence specter was checked using a confocal microscope (Zeiss LMS880, Carl Zeiss Microscopy GmBH, Iena, Germany). For temperature assays at 30 °C, we used an Axio Zoom V16 fluorescence stereomicroscope (Carl Zeiss Microscopy GmBH, Iena, Germany). Specific GFP fluorescence was detected using a short-pass filter (excitation filter, 470/40 nm; barrier filter, 525/50 nm). To detect all UV-fluorescent cell components including chlorophyll and GFP, a long-pass filter (excitation filter, 572/25 nm; barrier filter, 629/62 nm) was used. Images were obtained using a digital video camera (Axiocam 506 mono) coupled to the Axio Zoom microscope.

### 2.6. RNA Isolation and RT-PCR Analyses

For BPMV RNA detection, inoculated and systemic leaves from BPMV-inoculated plants were sampled at 7 and 14 or 21 dpi, respectively. Total RNA was extracted using the NucleoSpin RNA kit (Macherey-Nagel, Hœrdt, France). RNA concentrations were determined by measuring the absorbance at 260 nm on a NanoDrop 8000 (Thermo Fisher Scientific, Waltham, MA, USA) and integrity was checked by electrophoresis on a 1% agarose BET gel. cDNA was synthesized from 1 µg of total RNA using Reverse Transcription (RT) ImProm-II^TM^ enzyme (Promega Corp., Madison, USA) and Oligo-dT (Promega Corp., Madison, WI, USA) according to the manufacturer’s protocol and finally diluted 2.5 times in Milli-Q H_2_O.

Semi-quantitative RT-PCR was performed on 1 µL of diluted cDNA using GoTaq G2 Flexi (Promega Corp., Madison, USA) and using primers specific to BPMV RNA1, RNA2, *PvUBIQUITIN* (*PvUBI*, reference gene), and *PvINSULIN-DEGRADING ENZYME* (*PvIDE*) (reference gene; primers IDE-F 5′-GCAACCAACCTTTCATCAGC-3′ and IDE-R 5′-AGAAATGCCTCAACCCTTTG-3′), as described previously [41]. After either 20 cycles for primers RNA1 and RNA2 or 25 cycles for primers *PvUBI* and *PvIDE*, PCR products were analyzed by electrophoresis on a 2% agarose BET gel.

BPMV virus titer was estimated using a method based on a quantitative RT-PCR (RT-qPCR) analysis by determining the quantity of BPMV RNA1 and plant *PvIDE* mRNA, in four biological and three technical replicates (unless otherwise stated), in order to obtain a ratio of virus RNA to plant RNA. RT-qPCR protocol and analyses were performed as described in Richard et al. [45]. Briefly, RT-qPCR analysis was performed with a LightCycler^®^ 96 instrument in a volume of 15 µL reaction containing 2 µL of diluted cDNA, each specific primer with a final concentration of 0.1 µM, 7.5 µL of SYBR Green (LightCycler^®^ 480 SYBR Green I Master, Roche), and distilled water. The program used consisted of initial denaturation at 95 °C for 5 min and 50 cycles of 15 s of denaturation, 15 s of hybridization, and 15 s of elongation at 95, 60, and 72 °C, respectively. The results were analyzed using LightCycler^®^ 96 software version 1.1.

## 3. Results

### 3.1. R-BPMV-Mediated Resistance Is Associated with Local HR Lesions at 20 °C Where BPMV Is Able to Multiply in a First Step

**Figure 1:**
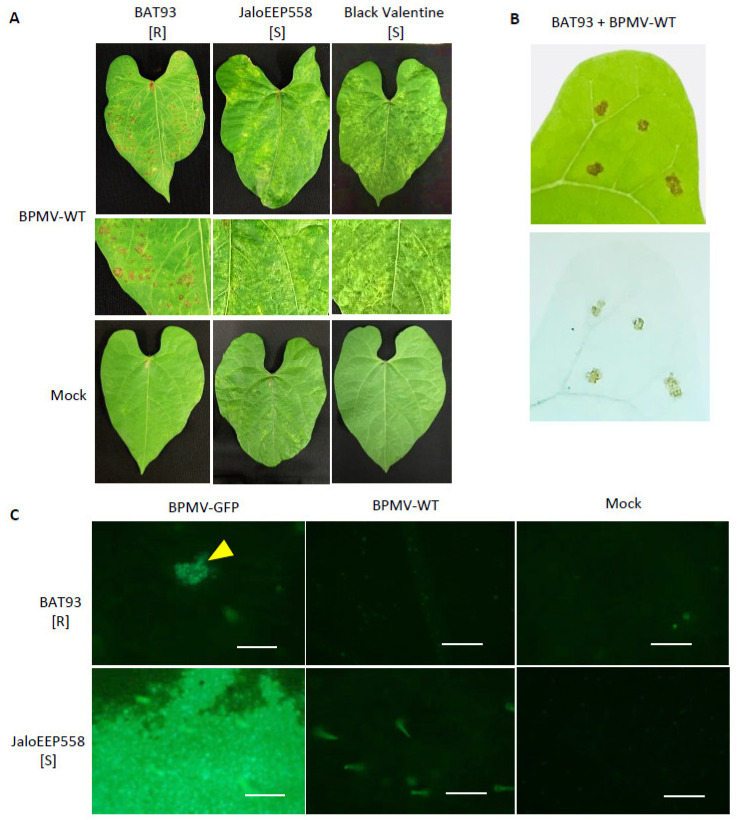
BPMV is able to multiply and move from cell-to-cell in the inoculated leaves of genotype BAT93 at 20 °C but infection is finally restricted by the induction of local Hypersensitive-Response (HR) lesions: (**A**) Representative pictures of local HR lesions on six BAT93-inoculated leaves of six different plants grown at 20 °C, 7 days post-inoculation (dpi) with BPMV-WT. JaloEEP558 and Black Valentine were used as susceptible control genotypes on which mosaic/mottling symptoms are visible on the inoculated leaves at 7 dpi with BPMV-WT. Mock was used as control. This experiment was performed at least 3 times with similar results. (**B**) Local HR lesions (top) and lesions visualized using trypan blue staining (bottom) on BAT93 leaves inoculated with BPMV-WT at 20 °C. Pictures were taken at 7 dpi. (**C**) Microscopic observations of GFP fluorescence in six inoculated leaves of six different plants of either BAT93 or JaloEEP558, both grown at 20 °C. Observations were made at 4 dpi. The BPMV-GFP construct expressing GFP as an individual protein processed from the RNA2 polyprotein was used as a reporter of BPMV infection. BPMV-WT and Mock were used as control. Yellow triangle indicates GFP foci. Autofluorescence is visible with BPMV-WT and Mock assays. All infection assays were made at 20 °C. Scale bars = 250 μm.

In previous work, we have shown that the Mesoamerican genotype BAT93 is resistant to BPMV and that resistance is controlled by a major gene, named *R-BPMV* [41]. In order to further characterize the BAT93 resistance phenotype, we performed inoculation assays using BPMV-WT and BPMV-GFP, a BPMV construct expressing GFP as a mature protein processed from the viral RNA2 polyprotein. We observed that at 7 dpi with BPMV-WT, a large number of macroscopic HR lesions developed in the inoculated leaves of BAT93 at 20 °C, whereas mosaic symptoms were visible in JaloEEP558 and Black Valentine, two susceptible genotypes lacking the *R-BPMV* gene (Figure 1A). Trypan blue staining assays at 7 dpi confirmed that HR lesions contained death cells (Figure 1B), thus attesting that these cells have undergone a cell death process induced by the *R-BPMV* gene. In order to study if BPMV can multiply in some individual cells before the establishment of HR lesions, we scanned the upper surface of BPMV-GFP-inoculated leaves of BAT93 at 4 dpi using an epifluorescence microscope. JaloEEP558 was used as a susceptible genotype. Interestingly, GFP fluorescence was detected in both BAT93 and JaloEEP558 at 4 dpi in inoculated leaves (Figure 1C). Widespread fluorescence was observed in JaloEEP558 leaves, whereas in BAT93, GFP fluorescence was found concentrated in a number of discrete areas that we will further call ‘GFP foci’ (Figure 1C). This suggests that resistance conferred by *R-BPMV* in BAT93 allows BPMV multiplication in small cell clusters but finally restricts virus spread, putatively by blocking virus cell-to-cell movement and/or inducing programmed cell death.

### 3.2. The R-BPMV Gene Segregates as an Incompletely Dominant Gene

To determine the inheritance of *R-BPMV*, we investigated the segregation of resistance versus susceptibility to BPMV in an F2 population of 60 plants derived from the cross BAT93 (resistant to BPMV) xJaloEEP558 (susceptible to BPMV). Each F2 plant was inoculated with the construct BPMV-GFP, and resistant/susceptible phenotypes were scored at 7 to 10 dpi. The observed segregation ratio was 56 resistant: 4 susceptible. The goodness-of-fit test indicated a significant deviation (χ^2^_1df_ = 10.74, *p* = 0.001) from the expected Mendelian ratio (3:1) with a deficit of susceptible phenotypes. Thus, the *R-BPMV* gene segregates as an incompletely dominant gene.

### 3.3. At 25 and 30 °C, R-BPMV Induces More Expanded Local HR Lesions

**Figure 2:**
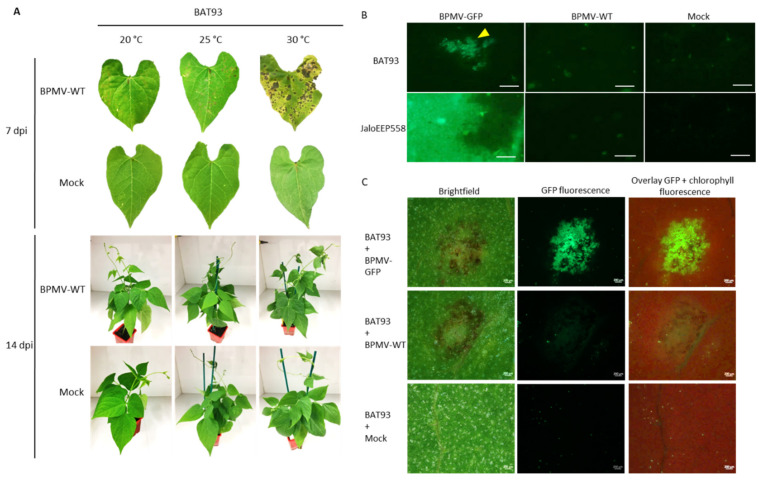
Elevated temperatures promote BPMV multiplication and cell-to-cell movement in the inoculated leaves of cv. BAT93 and larger local Hypersensitive-Response lesions are induced: (**A**) Representative pictures of local HR lesions on six BAT93-inoculated leaves from six different plants at 20 °C, 25 °C and 30 °C, at 7 days post-inoculation (dpi) with BPMV-WT (upper panel) and six whole plants of BAT93 at 20 °C, 25 °C and 30 °C, 14 dpi with BPMV-WT (lower panel). Mock was used as control. This experiment was performed at least 3 times with similar results. (**B**) Microscopic observations of BPMV-GFP accumulation in inoculated leaves of BAT93 and JaloEEP558 grown at 25 °C. The BPMV-GFP construct expressing GFP as an individual protein processed from the RNA2 polyprotein was used as a reporter of BPMV accumulation. BPMV-WT and Mock were used as control. All infection assays were made at 25 °C. Observations of GFP fluorescence (indicated by a yellow triangle) were made at 4 dpi and are representative of six BAT93-inoculated leaves sampled on six different plants. Scale bars = 250 μm. (**C**) Microscopic observations of BPMV-GFP accumulation in inoculated leaves of BAT93 grown at 30 °C. The BPMV-GFP construct expressing GFP as an individual protein processed from the RNA2 polyprotein was used as a reporter of BPMV accumulation. From left to right: brightfield, BPMV-GFP accumulation (green), overlay of chlorophyll fluorescence (red) and BPMV-GFP accumulation (green). BPMV-WT and Mock were used as control. All infection assays were made at 30 °C. Observations of GFP fluorescence were made at 4 dpi and are representative of six BAT93-inoculated leaves sampled on six different plants. Scale bars = 200 μm.

In previous work, we showed that *R-BPMV* is closely linked to the *I* gene at one end of chromosome 2 of *P. vulgaris* [41]. It is known that *I*-mediated resistance is associated with a systemic necrosis that may kill the host when infected by either BCMV above 28 °C or BCMNV regardless of the temperature [46,47,48]. Thus, the resistance phenotype conferred by the *I* gene in response to BCMV infection is temperature-dependent and switches at 28 °C from extreme resistance to systemic necrosis. In order to check if the resistance mediated by *R-BPMV* is dependent on temperature, we studied the resistance phenotype at 25 and 30 °C. We first performed inoculation assays using BPMV-WT on the resistant genotype BAT93. We observed that the resistance phenotype is expressed as local HR lesions in the inoculated leaves at all tested temperatures but that HR lesions become more expanded as the temperature increases (Figure 2A). Nevertheless, no vascular systemic necrosis appears at 14 dpi in apical parts of the inoculated plants (Figure 2A). Moreover, no viral symptoms were visible in these apical parts (Figure 2A) and no viral RNAs were detected in systemic leaves of BAT93-inoculated plants at all temperatures (Figure S1). Thus, in our pathosystem, BPMV was blocked in the inoculated leaves and could not spread systemically at all tested temperatures (20, 25, and 30 °C). In conclusion, no switch of phenotype was observed in BAT93 when inoculated with BPMV at either 25 or 30 °C.

In order to assess if larger HR lesions at elevated temperature were correlated with a more expanded multiplication area of BPMV, we scanned the upper surface of the BPMV-GFP-inoculated leaves of BAT93 at 4 dpi using an epifluorescence microscope (25 °C) or Axio Zoom microscope (30 °C). JaloEEP558 was used as a susceptible genotype [41]. As expected, at 4 dpi, many GFP foci were detected on the upper surface of BPMV-GFP inoculated leaves of BAT93 at 25 and 30 °C (Figure 2B,C). Overall, the GFP foci approximately doubled when shifting from 20 to 25 °C and from 25 to 30 °C. Thus, BPMV is still able to multiply and to move from cell-to-cell in BAT93 at 25 and 30 °C. Consequently, the plant defense response mediated by *R-BPMV* seems to become less efficient when temperature increases from 20 to 30 °C as larger GFP foci are observed.

### 3.4. The Resistance Mediated by R-BPMV Is Heat-Stable up to 35 °C in BAT93, But Local HR Lesions Are Replaced by Chlorotic Lesions

**Figure 3:**
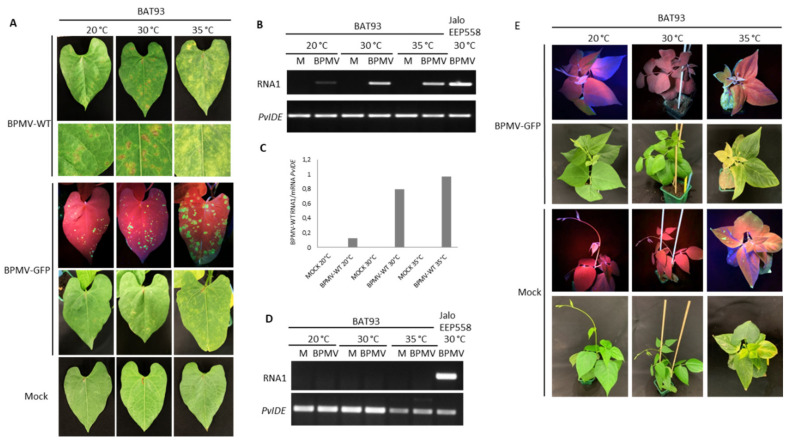
The *R-BPMV* gene of BAT93 is still efficient to confer BPMV resistance at 35 °C: (**A**) Representative pictures of BAT93-inoculated leaves at 20 °C, 30 °C and 35 °C, 7 days post-inoculation (dpi) with BPMV-WT (upper panel; six plants), BPMV-GFP (middle panel; six plants) and Mock (lower panel; six plants). BPMV-GFP was detected under UV light. This experiment was performed at least three times with similar results. (**B**) Semi-quantitative RT-PCR of BPMV RNA1 (upper panel) in BAT93-inoculated leaves. Plants were inoculated with either mock (M) buffer or BPMV-WT (BPMV) at 20 °C, 30 °C and 35 °C. *PvIde* was used as an internal control. Total RNA was extracted at 7 dpi from a pool of three BAT93-inoculated leaves sampled on three different plants. A sample of inoculated leaves of JaloEEP558 at 30 °C was taken as positive control for PCR amplification. (**C**) Quantification of BPMV titer at 7 dpi in BPMV-WT-inoculated leaves of BAT93 by calculation of the relative ratio of BPMV RNA1 to plant mRNA of *PvIde* using a quantitative RT-PCR procedure. Data are mean ratios of pools of three BAT93-inoculated leaves sampled on three different plants. (**D**) Semi-quantitative RT-PCR of BPMV RNA1 (upper panel) in systemic leaves of BAT93 plants inoculated with either mock (M) buffer or BPMV-WT (BPMV) at 20 °C, 30 °C and 35 °C. *PvIde* was used as an internal control. Total RNA was extracted at 14 dpi from a pool of two systemic leaves (third and fourth trifoliate) sampled on two-three different plants of BAT93. A sample of inoculated leaves of JaloEEP558+BPMV-WT at 30 °C was taken as positive control for PCR amplification. This experiment was performed twice with similar results. (**E**) Representative pictures of six whole plants of BAT93 at 20 °C, 30 °C and 35 °C, 11 dpi with either BPMV-GFP (upper panel) or mock buffer (lower panel). BPMV-GFP was detected under UV light.

It is known that some dominant *R* genes against viruses are overcome at elevated temperatures (e.g., at 28 °C for *N* or at 30 °C for *Tsw*) [22,23,49], meaning that the resistance status of the genotype is broken and systemic infection of upper leaves occurs, leading to a susceptible state. We found that *R-BPMV*-mediated resistance was still efficient at temperatures up to 30 °C, but we wanted to test whether higher temperatures (35 °C) could break the resistance conferred by *R-BPMV*. For this purpose, we inoculated BAT93 plants with BPMV-WT and BPMV-GFP and placed the plants at constant 35 °C. As expected, at 7 dpi, HR lesions developed on inoculated leaves of BAT93 + BPMV-WT at 30 °C, and GFP fluorescence was macroscopically detected on inoculated leaves of BAT93 + BPMV-GFP (Figure 3A). By contrast, at 35 °C, only chlorotic lesions (i.e., light-green or yellow local lesions with no visible cell death/necrosis) were visible at 7 dpi on inoculated leaves of BAT93 + BPMV-WT. This appears to correspond to large infection areas, as confirmed by expanded GFP foci visible in inoculated leaves of BAT93 + BPMV-GFP (Figure 3A). Quantification of virus titer by RT-PCR in the inoculated leaves of BAT93 confirmed a higher accumulation of viral RNAs at 35 °C compared to 30 and 20 °C (Figure 3B,C).

To ensure that no virus moved systemically, we studied the systemic leaves of BAT93 plants grown at 35 °C and inoculated with either BPMV-WT or BPMV-GFP. No viral RNAs were detected in systemic leaves at 7 dpi, and no GFP fluorescence was visible (Figure 3D,E), attesting to the fact that although local infection is of higher intensity, the systemic infection is prevented. Furthermore, no systemic necrosis occurred (Figure 3E). In parallel, we used the susceptible genotype Black Valentine as a control to assess if at 35 °C, BPMV-GFP conserved infectiousness and capacity for systemic movement. We observed that GFP fluorescence was widespread in all systemic leaves of Black Valentine plants at 35 °C 7 dpi, including stems, while no GFP was detected in the systemic leaves of plants grown at 20 °C (Figure S2). Overall, our results demonstrate that *R-BPMV* is heat-stable up to 35 °C. The *R-BPMV*-mediated phenotype is expressed as chlorotic lesions in the inoculated leaves, and no symptoms are detected in the systemic parts.

### 3.5. Elevated Temperatures Boost BPMV Infection in Susceptible Genotypes

**Figure 4:**
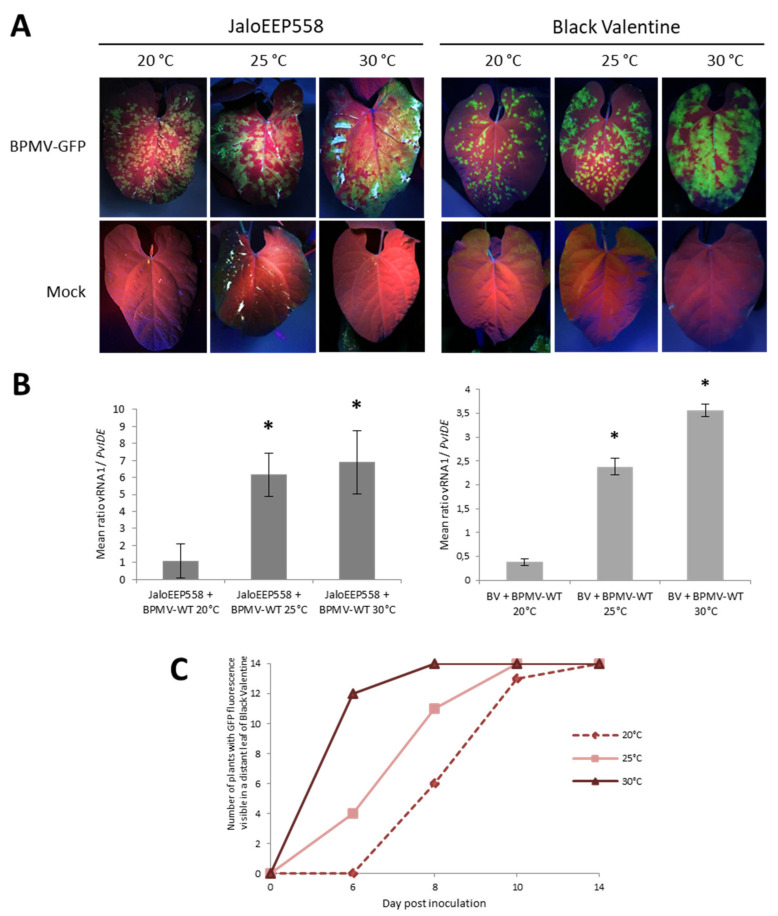
Elevated temperatures promote BPMV infection in the inoculated leaf and BPMV systemic spreading in whole plants of two susceptible genotypes of *P. vulgaris*: (**A**) Representative pictures of four inoculated leaves from four different plants of either JaloEEP558 or Black Valentine (left and right panel, respectively) at 20 °C, 25 °C and 30 °C, 7 days post-inoculation (dpi) with BPMV-GFP (upper panel) and Mock (lower panel). BPMV-GFP was detected under UV light. This experiment was performed at least three times with similar results. (**B**) Quantification of BPMV titer at 7 dpi in four BPMV-WT-inoculated leaves of four different plants of JaloEEP558 or Black Valentine (BV) by calculation of the relative ratio of BPMV RNA1 to plant mRNA of *PvIde* using a quantitative RT-PCR procedure. Asteriks indicate significant differences between the tested temperature and 20 °C, the control temperature (*t*-test, *p*-value < 0,05). No significant difference of viral titer was found between the two elevated temperatures 25 °C and 30 °C. Data are mean ratios ± SD of four biological replicates. (**C**) BPMV-GFP systemic spreading in whole plants of Black Valentine at 20 °C, 25 °C, and 30 °C. The graph represents the number of plants with GFP fluorescence visible in a distant leaf scored at four dates after inoculation with BPMV-GFP: 0, 6, 8, 10, and 14 dpi on a total of fourteen plants per temperature assay. BPMV-GFP was detected under UV light. This experiment was performed twice with similar results.

In the resistant genotype BAT93, elevated temperatures (especially 35 °C) promoted viral replication and cell-to-cell movement in the inoculated leaves (Figure 3A). Therefore, we investigated the effect of elevated temperatures on BPMV infection in the two susceptible genotypes, JaloEEP558 and Black Valentine. We followed the BPMV-GFP accumulation by visual inspection of plants inoculated with BPMV-GFP grown either at 25 or 30 °C. Compared to control plants at 20 °C, GFP foci at 25 and 30 °C on inoculated leaves of JaloEEP558 and Black Valentine at 7 dpi were more expanded and confluent on the upper side of the leaves (Figure 4A). Quantification of BPMV RNA1 in leaves inoculated with BPMV-WT confirmed the higher accumulation of BPMV at 25 and 30 °C compared to 20 °C (Figure 4B). More precisely, significantly more RNAs were detected at 25 and 30 °C compared to 20 °C (~6 fold- and ~7–9-fold increase, respectively) (Figure 4B). Consequently, BPMV replication and cell-to-cell movement are more efficient from 0 to 7 dpi in the susceptible genotypes JaloEEP558 and Black Valentine at elevated temperature (25 and 30 °C) compared to 20 °C.

Furthermore, the effect of temperature on the rate of systemic spreading of BPMV-GFP was investigated by scoring the date of appearance of GFP foci on systemic leaves of Black Valentine plants at 20, 25, and 30 °C between 6 and 14 dpi. From 6 to 10 dpi, the systemic spreading is faster at 30 °C compared to 25 °C, and at 25 °C compared to 20 °C (Figure 4C). At 10 dpi, GFP foci in systemic leaves of plants at 20 °C were tiny, whereas GFP fluorescence was widespread at 25 and 30 °C (Figure S3A). At 14 dpi, all plants at 20, 25, and 30 °C were systemically infected by BPMV-GFP (Figure 4C). High levels of fluorescence were visible in all inoculated parts of plants grown at 25 and 30 °C (Figure S3B). These results confirm that BPMV systemic movement becomes faster when temperature increases from 20 to 30 °C, resulting in a higher disease incidence in susceptible genotypes.

### 3.6. BT-1 and BT-2, Two Near Isogenic Lines for the I. locus, Are Both Resistant to BPMV Systemic Movement

The *R-BPMV* gene is closely linked to *I*, a famous locus conferring a broad-spectrum resistance to at least ten different *Potyviruses* (reviewed in [38]) and several *Comoviruses*. In this work, we showed that *R-BPMV* segregates as an incompletely dominant gene. BT-1 (resistant to BCMV, *I*/*I*) and BT-2 (susceptible to BCMV, *i*/*i*) are two near-isogenic lines created to study the *I* locus [42]. We tested them to decipher the resistance mechanism mediated by the *R-BPMV* gene at the tissue and cell levels, as done for the *I* locus [50,51,52]. We challenged BT-1 and BT-2 for their resistance/susceptibility to BPMV at three temperatures: 20, 25, and 30 °C.

**Figure 5:**
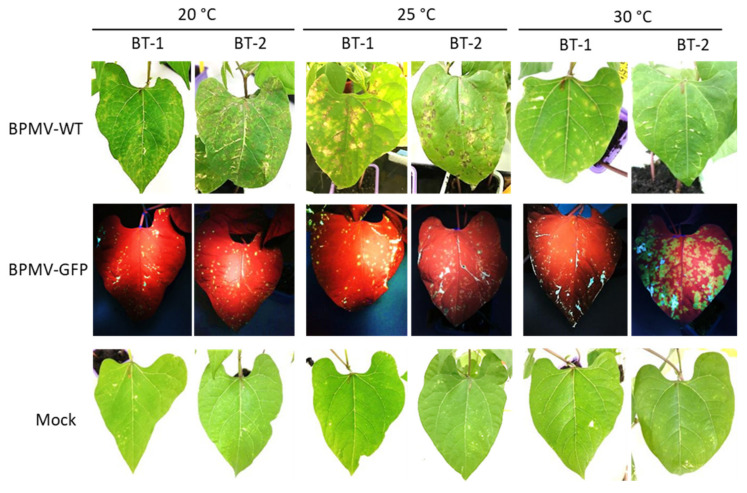
BT-1 and BT-2, two Near Isogenic Lines for the *I* locus are both resistant to BPMV systemic movement: Representative pictures of four BT-1- and four BT-2-inoculated leaves (sampled on four different plants) grown respectively at 20 °C, 30 °C and 35 °C, 7 days post-inoculation (dpi) with BPMV-WT (upper panel), BPMV-GFP (middle panel), and Mock (lower panel). BPMV-GFP was detected under UV light. This experiment was performed twice with similar results.

At 7 dpi, local HR lesions developed on inoculated leaves of BT-1 and BT-2 at 20 and 25 °C (Figure 5). As in BAT93, these local HR lesions corresponded to infection areas in which BPMV can first multiply, since small GFP foci were visible after inoculation with BPMV-GFP in BT-1 and BT-2 (Figure 5). Thus, both BT-1 and BT-2 behave as resistant genotypes to BPMV at 20, 25, and 30 °C, and this is strengthened by the observation that no BPMV RNAs were detected in systemic leaves at 21 dpi (Figure S4) as well as no viral symptoms or systemic necrosis (data not shown). In conclusion, BT-1 and BT-2 will not be useful tools to dissect *R-BPMV*-mediated resistance at the cellular level.

## 4. Discussion

In this work, we studied the behavior of the *R-BPMV*-mediated resistance to BPMV in *P. vulgaris* at normal (20 °C) and elevated temperatures (25, 30, and 35 °C). Strikingly, we found that *R-BPMV*-mediated resistance is heat-stable up to constant 35 °C at the whole plant level (Figure 3), in the sense that no systemic infection is observed and BPMV remains confined to the inoculated leaf.

This is in agreement with results obtained for other plant–virus pathosystems for which heat-stable *R* genes have been described. In pepper, different natural allelic variants from the same gene may have different behaviors as reported for the *L^1c^* allele, which is heat-stable at 30 °C, whereas other alleles (*L^1^*) are not [53]. More recently, the potato NLR *Rx1* gene was reported to confer a temperature-insensitive resistance to PVX in *Nicotiana benthamiana* that is not overcome at temperatures up to 32 °C, a temperature at which the virus was no longer infectious [24]. Additional heat-stable *R*-gene-mediated resistance has also been reported for pathogens other than viruses. For instance, the tomato *Mi-9* gene confers a heat-stable resistance to root-knot nematodes (*Meloidogyne* sp.) [54,55], despite being homologous to the NLR *Mi-1*, which is heat-sensitive [56]. In pepper, several heat-stable *R* genes against *Meloidogyne* species, named *Me*, have been reported [57,58]. In *Solanum* species, *Ry_sto_* and *Ry_chc_ R* genes present in *S. stoloniferum* and *S. chacoense*, respectively, confer extreme resistance to the *Tobacco veinal necrosis strain* of PVY (PVY^N^) and are functional at both low (16–20 °C) and elevated temperatures (above 24 °C) [59,60].

On the other hand, temperature sensitivity has more often been reported in the literature. In tobacco, the NLR *N* is unable to confer resistance to the *Tobacco mosaic virus* (TMV) above 28 °C [22,49]. Accordingly, the *Capsicum* sp. NLR *Tsw* fails to trigger resistance to the *Tomato spotted wilt virus* (TSWV) at 32 °C and above [23,61]. In potato, some *R* genes conferring HR to PVY including *Ny* in *Solanum sparsipilum* and *S. sucrense*, or *Ny-1* in *S. tuberosum* cv. Rywal, confer resistance only at low temperatures (16–20 °C), whereas at higher temperatures (24–28 °C), resistance is inhibited and PVY infects plants systemically [60]. Concerning the other kinds of pathogens, temperature sensitivity has been reported for the NLR *Mi-1* in tomato (resistance to *Meloidogyne incognita*, [62]), for the NLR *Bs2* in pepper (resistance to *Xanthomonas axonopodis* pv. *vesicatoria*, [63]), the non-NLR *Cf-4* and *Cf-9* genes (resistance to *Cladosporium fulvum*, [64,65]), as well as for many *R* genes against bacteria in Arabidopsis (e.g., *RPS2* and *RPM1* resistance to *Pseudomonas syringae*, [66]; *RPS2* and *ZAR1* resistance to *P. syringae*, [67]; *RPS4/RRS1-R* resistance to *Ralstonia solanacearum*, [68]; and *SNC1* and *RPS4* resistance to *P. syringae* and *Peronospora parasitica*, [69,70,71]). All these data converge to assess that temperature sensitivity of resistance is widespread, whereas heat-stable *R* genes are scarce.

Mechanisms underlying heat sensitivity are poorly understood. Zhu et al. [70] demonstrated that the R proteins, involved in the recognition of pathogen effectors, can be themselves the causal temperature-sensitive component in defense responses. Indeed, autoimmunity-associated mutations (e.g., mutants in which ETI and spontaneous cell death are constitutively activated) in SNC1, an Arabidopsis NLR-homolog, cause increased SNC1 nuclear localization at 22 °C. In contrast, at 28 °C, the reduced nuclear localization is associated with the suppression of autoimmune phenotypes. Similarly, nuclear localization of the R protein N of tobacco was observed after recognition of the TMV coat protein at 22 but not at 28 °C [70]. When the two SNC1 mutations were introduced into the *N* gene, resistance was effective at elevated temperature, thus suggesting that these mutations may prevent the temperature-sensitive conformational loss of function of the NLR N [70] and maintaining its interaction with the TMV coat protein [22,49]. It is not excluded that such differential point mutations could exist between *R-BPMV* and *I* genes, and one way to confirm this hypothesis would be to clone their corresponding gene sequences. Other putative mechanisms of temperature sensitivity have been proposed such as homeostasis of R-interacting chaperons, reduced R protein amounts resulting in a lower R activity, and deregulation of nucleo-cytoplasmic localization of R proteins (reviewed in [18]). More recently, the implication of the methionine cycle [72] and deregulation of the intercellular communication via plasmodesmata [73] have also been supposed to influence virus spread within their hosts.

Our results show that the major gene *R-BPMV* in *P. vulgaris* genotype BAT93 confers resistance to BPMV by inducing local HR lesions at 20 °C, where BPMV is able to multiply in a first step (Figure 1). HR lesions have also been observed at 25 and 30 °C 7 dpi (Figure 2). Interestingly, at 7 dpi, we observed larger HR lesions at 25 and 30 °C compared to 20 °C, and this observation was correlated with (i) more expanded multiplication areas of BPMV visualized as green fluorescence areas in BPMV-GFP infected leaves at 4 dpi (Figure 2) and (ii) a rising virus titer in the inoculated leaves at 7 dpi (Figure 3B,C). Consequently, we propose that the higher levels of BPMV accumulation and cell-to-cell spreading in the primary sites of infection could be attributed to a delayed defense response when temperature increases from 20 to 30 °C. As biotrophic pathogens, viruses mainly induce defense responses regulated by salicylic acid (SA) signaling [74], and SA was shown to be a key component that orchestrates the events restricting viral spread in HR [75,76]. Moreover, HR-mediated resistance against *Turnip crinkle virus* (TCV) was impaired in Arabidopsis *eds5* and *sid2* SA-deficient mutants without affecting HR cell death [77]. Lukan et al. [78] showed that *Potato virus Y* (PVY, *Potyvirus*) spread is even faster in SA-depleted plants (*NahG*-potato transgenic plants) with rapid lesion expansion. Interestingly, the expression of SA-dependent responses was reduced at elevated temperature in tobacco and potato plants infected with *Cucumber mosaic virus* (CMV) and PVY, respectively [79,80]. Whether the SA accumulation or other defenses are inhibited or downregulated at elevated temperature needs further work in our pathosystem. Moreover, it has been shown that virus resistance is uncoupled from cell death and that these two events are likely independent [77,78,81,82,83,84,85,86,87]. For example, in potato plants bearing the temperature-sensitive *Ny-1 R* gene against PVY, temperature shift assays from 22 to 28 °C induced the detection of PVY in infected cells outside the cell death zone [78]. Nevertheless, the molecular mechanisms restricting both virus spread and cell death remain unknown [88].

Interestingly, at 35 °C, HR lesions that developed on BPMV-inoculated leaves of BAT93 were replaced by chlorotic lesions, while the *R-BPMV*-mediated resistance remains functional at the whole-plant level (Figure 3). We confirmed that BPMV is still infectious and capable of systemic movement at 35 °C (Figure S2). Thus, it seems that cell death is completely abolished at 35 °C, whereas the local defense response is still efficient to block BPMV systemic infection (Figure 3E). The reduction of cell death at elevated temperature has already been reported in soybean (*Glycine max*) infected by strains G1 and G7 of *Soybean mosaic virus* (SMV) [89]. Indeed, a stem-tip necrosis (STN) develops in several soybean cultivars carrying the *R* genes *Rsv1* and *Rsv1-n*. The STN in genotype V262 induced by strain G1 and in genotype V94-3971 by strain G7 developed at 10, 15, 20, 25, and 30 °C, on 44, 41, 14, 8, and 8 days after inoculation, respectively. By contrast, at 35 °C, no STN reaction and, consequently, no cell death was induced by any strain. Interestingly, cell death reduction at elevated temperature has also been reported for plant–bacteria interactions. AvrRpt2-mediated cell death in Dex-AvrRpt2 plants was significantly reduced at 28 °C and was almost completely abolished at 32 °C [66]. Interestingly, the expression of the *WORKY46* transcription factor acting as an upstream regulator of SA metabolism was also temperature-sensitive with a peak at 16 °C and a decrease at temperatures above 28 °C. In the same way, AvrRpm1- and AvrB-mediated cell death was significantly attenuated at elevated temperatures (32 °C) [66]. Thus, the data indicate that elevated temperature could suppress ETI signaling in *R*-gene-mediated response.

We previously showed that *R-BPMV* is present in genotype BAT93 of *P. vulgaris* and that this gene is genetically linked to the *I* locus on chromosome 2 [41,90]. The *I* locus is a broad-spectrum resistance locus that confers resistance to BCMV, BCMNV [91,92], and nine other potyviral species (*Watermelon mosaic virus-2* [93,94], *Cowpea aphid-borne mosaic virus* [93,95], *Soybean mosaic virus* [96,97], *Peanut mottle virus* [98,99], *Zucchini yellow mosaic virus, Thaïland passiflora mosaic virus*, and *Passionfruit woodiness virus-K* [100], BYMV, and ClYVV [101,102]). Thus, we hypothesized that *R-BPMV* and *I* could correspond to the same gene with pleiotropic effects and if so, one important question would be: do *R-BPMV*- and *I*-mediated resistance have the same behavior regarding temperature?

**Figure 6:**
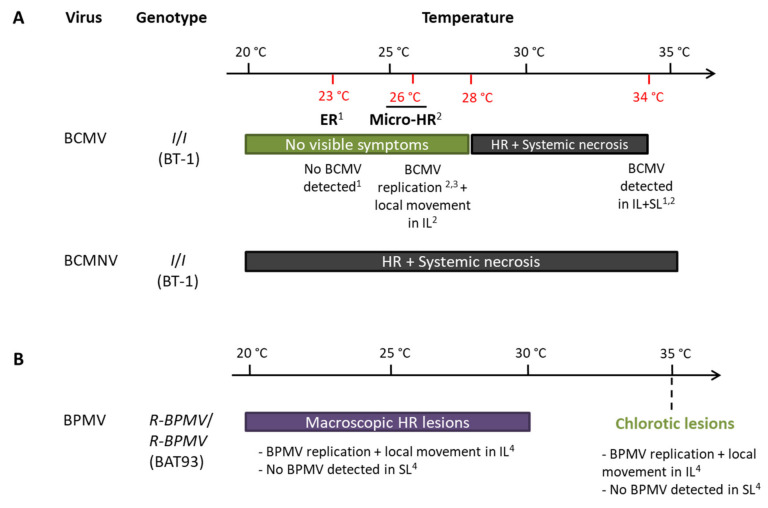
Resistance phenotypes of *R-BPMV*- and *I*-derived resistances do not share the same features regarding temperature: (**A**)Potyviruses *Bean common mosaic virus* (BCMV) and *Bean common mosaic necrosis virus* (BCMNV); (**B**)Comovirus *Bean pod mottle virus* (BPMV) Abreviations: ER: Extreme resistance, HR: Hypersensitive Reaction, IL: inoculated leaf, Micro-HR: microscopic Hypersensitive Reaction, SL: systemic leaf. References: 1: Collmer et al. 2000 (whole-plant study); 2: Cadle-Davidson and Jahn 2006 (whole-plant study); 3: Cadle-Davidson and Jahn 2005 (BCMV transfection in protoplasts); 4: this study.

*I* was reported to be temperature-sensitive for BCMV resistance [47,48]: an extreme resistance or micro-HR phenotypes are observed on inoculated leaves below 28 °C, and BCMV replication was effective [51], but above 28 °C, the ER evolves into a systemic necrosis [48,50,52] (Figure 6). However, for BCMNV, a local HR in inoculated leaves evolves into a systemic necrosis regardless of temperature [46,47,48] (Figure 6). With BPMV, we observed local HR lesions in inoculated leaves of BAT93 at 20, 25, and 30 °C at 7 dpi in which BPMV is able to multiply in a first step (Figure 1 and Figure 2). By contrast, at 35 °C, chlorotic lesions develop in inoculated leaves with no BPMV detected in the systemic leaves (Figure 3A), although BPMV is still infectious at 35 °C in the susceptible genotype BV (Figure S2) with a higher rate of systemic spread at 25, 30, and 35 °C compared to 20 °C (Figures S2 and S3). Thus, our results highlight a switch in the resistance phenotype mediated by *R-BPMV* that occurs between 30 and 35 °C (Figure 6). Compared to the *I* gene, this is a significant difference of behavior of the two genetically linked-resistance genes, since *I* induces local HR and systemic necrosis from 28 °C with BCMV detected in the inoculated leaves and systemic leaves [50,52] (Figure 6).

Overall, we show here that *R-BPMV*- and *I*-mediated resistance phenotypes do not behave in the same way at an elevated temperature. Thus, although genetically tightly linked, *BPMV* and *I* could correspond to two different genes. Another hypothesis is that resistance to BPMV and BCMV is controlled by the same gene but that the downstream signaling cascades differ, explaining the two contrasting kinds of resistance phenotypes. Neither *I* nor *R-BPMV* has been cloned but both were hypothesized to encode NLR receptors [90,91,103]. Consistent with this hypothesis, the resistance phenotypes are expressed for *I* as either extreme resistance or systemic necrosis or for *R-BPMV* as local HR, which are hallmarks of ETI mediated by NLR receptors. Moreover, Vallejos et al. [91] reported an over-expression of several NLR genes of the *I* region after BCMV or BCMNV inoculations, suggesting a role of NLR genes in the response to BCMV/BCMNV.

Finally, it is worth noting that both *R-BPMV* (our study) and *I* segregate as incompletely dominant genes [50,51,91]. Indeed, when investigating the inheritance of *R-BPMV* in an F2 population of 60 plants, we found a deficit of susceptible phenotypes (56 resistant: 4 susceptible) and thus, a significant deviation from the expected Mendelian ratio (3:1). A similar segregation deviation was reported for the *I* gene in an F2 population obtained from the cross Calima (susceptible Andean genotype) × Jamapa (resistant Mesoamerican genotype) [91]. Vallejos et al. [91] suggested that this phenomenon was a consequence of the partial compatibility of the Andean and Mesoamerican genomes interacting in this type of crosses [91].

To study the effect of elevated temperatures in a *P. vulgaris*-BPMV compatible interaction, we performed experiments on two BPMV-susceptible genotypes *P. vulgaris* cv. Black Valentine and cv. JaloEEP558. We reported that in both genotypes, the area of primary infection sites at 7 dpi was larger on BPMV-GFP-inoculated leaves at 25 and 30 °C compared to 20 °C, and these results were correlated with a higher virus titer in these leaves (Figure 4A,B). Moreover, in Black Valentine, the virus spreads systemically even more rapidly when the temperature increases (Figure 4C). Overall, our results show a positive effect of temperature elevation on the level of susceptibility in two susceptible genotypes. Our results are in agreement with several other studies on different plant–virus interactions in compatible contexts. For example, Chung et al. [29] investigated the effects of different temperature regimes on the speed of systemic spread after inoculation of *Turnip mosaic virus* (TuMV) in Chinese cabbage. It took 48 days for systemic infection to occur at 13 °C but only 6 days at 22–33 °C. Likewise, in *Solanum tuberosum*, plants became 100% systemically infected at 24 and 28 °C, while at 20 °C, only 20% of the plants were systemically infected [26,27]. Nancarrow et al. [27] studied the effects of elevated (10–21 °C, night/ day) or ambient (5–16 °C, night/day) temperature winter growing season regimes on wheat plants infected with *Barley yellow dwarf virus* (BYDV, *Luteovirus*). Infected plants grown under an elevated temperature developed virus symptoms earlier and had higher virus titers than plants grown at an ambient temperature. In potato, susceptibility to PVY was dramatically increased in systemic infected leaves at higher temperatures [80]. In cassava, geminiviruses responsible for cassava mosaic disease cause more symptoms and have higher viral titer at 25 °C compared to 30 °C [32]. Nevertheless, other studies have shown opposite results, showing a negative effect on the level of susceptibility in susceptible genotypes. In *N. benthamiana*, plants infected with the potyviruses PVY and *Potato virus A* had fewer symptoms and reduced CP protein accumulation at 20 °C compared to 30 °C [104]. In the same way, Del Toro et al. [30] worked in *N. benthamiana* infected with PVY and PVX and showed attenuated symptoms for PVY and no symptoms for PVX at 30 °C compared to 25 °C. Aguilar et al. [105] described that elevated temperatures decrease both virulence and virus titers in the synergistic infection PVX/*Plum pox virus* (PPV) in *N. benthamiana*. Importantly, the RNA silencing machinery was shown to be more active at elevated temperatures in several plants including cassava and *N. benthamiana* [32,106], suggesting that more efficient RNA silencing is responsible for impaired viral replication (or the viral RNA suppressor is less active). To conclude, all these studies demonstrate that depending on the virus and plant host, elevated temperature may either increase or decrease virus susceptibility. To investigate this differential impact, further studies are needed on other pathosystems in the future. Moreover, molecular mechanisms underlying the differential susceptibility to viruses must be investigated in more depth, especially concerning the implication of the RNA silencing pathway in pathosystems for which viral infection is boosted at elevated temperature such as *P. vulgaris*-BPMV.

Global warming leads to rising temperatures, which alter plant–virus interactions [73], potentially inducing yield losses and decreasing quality of crop productions [107]. It is therefore timely to study the effect of elevated temperatures on plant responses to virus infections. Importantly, future experiments on the effect of temperature on plant–virus interactions will need to consider the dynamic nature of field conditions, with diurnal fluctuating temperature cycles and heat waves such as those experienced by crops in the field. As performed in Nancarrow et al. [27], growing chambers will need to more closely resemble the dynamic conditions to which plants and pathogens are subjected in nature so that field trials can be mimicked. After studying the effect of a single environmental factor in order to have a specific response, it will be important to test the effect of several combined environmental factors (elevated temperature + high CO_2_ for example) to analyze whether their effects are additive or antagonistic. In C3 plants like *P. vulgaris*, high CO_2_ improves photosynthesis and activates hormonal pathways, whereas high temperatures decrease the efficiency of Rubisco and the solubility of CO_2_. Consequently, combined high CO_2_ and elevated temperatures may induce antagonistic effects. At the international level, little work has been published on the combined effects of elevated temperatures and high CO_2_ on plant–virus interactions (reviewed in [20]). Del Toro et al. [108] focused on combined climate conditions (CCC, elevated temperature (30 versus 25 °C) and high CO_2_ level (970 versus 405 ppm)) by studying CMV, PVY, and PVX in the model plant *N. benthamiana*. They showed that viral titers in systemic leaves under CCC showed no significant differences at 7 and 12 dpi for CMV. Conversely, viral titers of PVY and PVX were significantly lower under CCC compared to the standard condition at both 7 and 12 dpi. In the case of PVY, viral titer was lower at 12 dpi compared to 7 dpi under CCC, and the contrary was observed for PVX [108]. In potato (*S. tuberosum*), Chung et al. [109] demonstrated that *Potato leaf roll virus* (PLRV) RNA accumulated at higher levels, and larger numbers of potato plants were infected by PLRV under combined high CO_2_ levels (940 ppm) and elevated temperature (30 °C). More recently, Aguilar et al. [110] studied a multifactorial system combining biotic (virus and bacteria) and abiotic (drought) stresses in *A. thaliana* (compatible context) and *N. benthamiana* plants (incompatible context). They demonstrated that infection by the PVX/PPV virus combination induced resistance to the bacteria *P. syringae* pv. *tomato* and to drought in both compatible and incompatible host–bacteria interactions. However, combined high CO_2_ levels (970 ppm) and elevated temperature (30 °C) negatively affected resistance to *Pst* and to drought induced by a virus infection, and this correlated with diminished H_2_O_2_ production, decreased expression of defense genes, and a drop in virus titers.

## 5. Conclusions

To conclude, our study gives a first insight into the impact of elevated temperature on the level of resistance/susceptibility to viruses in common bean. Indeed, natural genetic resistance is often the most effective and environmentally friendly way of controlling plant diseases, in particular against a virus where no chemical alternative is available. This research needs to be continued in order to decipher the underlying cellular and molecular mechanisms of heat tolerance and to engineer robust thermostable resistances in the context of global warming.

## Data Availability

Data sharing is not applicable to this article.

## Supplementary Materials

The following are available online at https://www.mdpi.com/article/10.3390/v13071239/s1, Figure S1, Semi-quantitative RT-PCR showing that *P. vulgaris* cv. BAT93 is resistant to BPMV systemic infection; Figure S2, BPMV is still infectious at 35 °C and spreads systemically in the susceptible genotype *P. vulgaris* cv. Black Valentine; Figure S3, Elevated temperatures promote BPMV spreading in the systemic leaves and in whole plants in the susceptible genotype *P. vulgaris* cv. Black Valentine; Figure S4, Semi-quantitative RT-PCR showing that *P. vulgaris* cv. BT-1 and BT-2, two Near Isogenic Lines for the *I* locus, are both resistant to BPMV systemic infection.

## Abreviations

Avr factor	avirulence factor
CO_2_	carbon dioxide
dpi	day(s) post-inoculation
ETI	effector-triggered immunity
HR	hypersensitive reaction
NLR	nucleotide-binding domain leucin-rich repeat containing receptors
ppm	parts per million
*R*	resistance gene/protein
