# Supplementary material for: R-BPMV-Mediated Resistance to Bean pod mottle virus in Phaseolus vulgaris L. Is Heat-Stable but Elevated Temperatures Boost Viral Infection in Susceptible Genotypes
Source: Viruses. 2021 Jun 26;13(7):1239. doi: 10.3390/v13071239 (PMC8310253; doi:10.3390/v13071239)
Supplement: Supplementary file 1 [file viruses-13-01239-s001.zip › viruses-1234162-supplementary.pptx]

## Slide 1
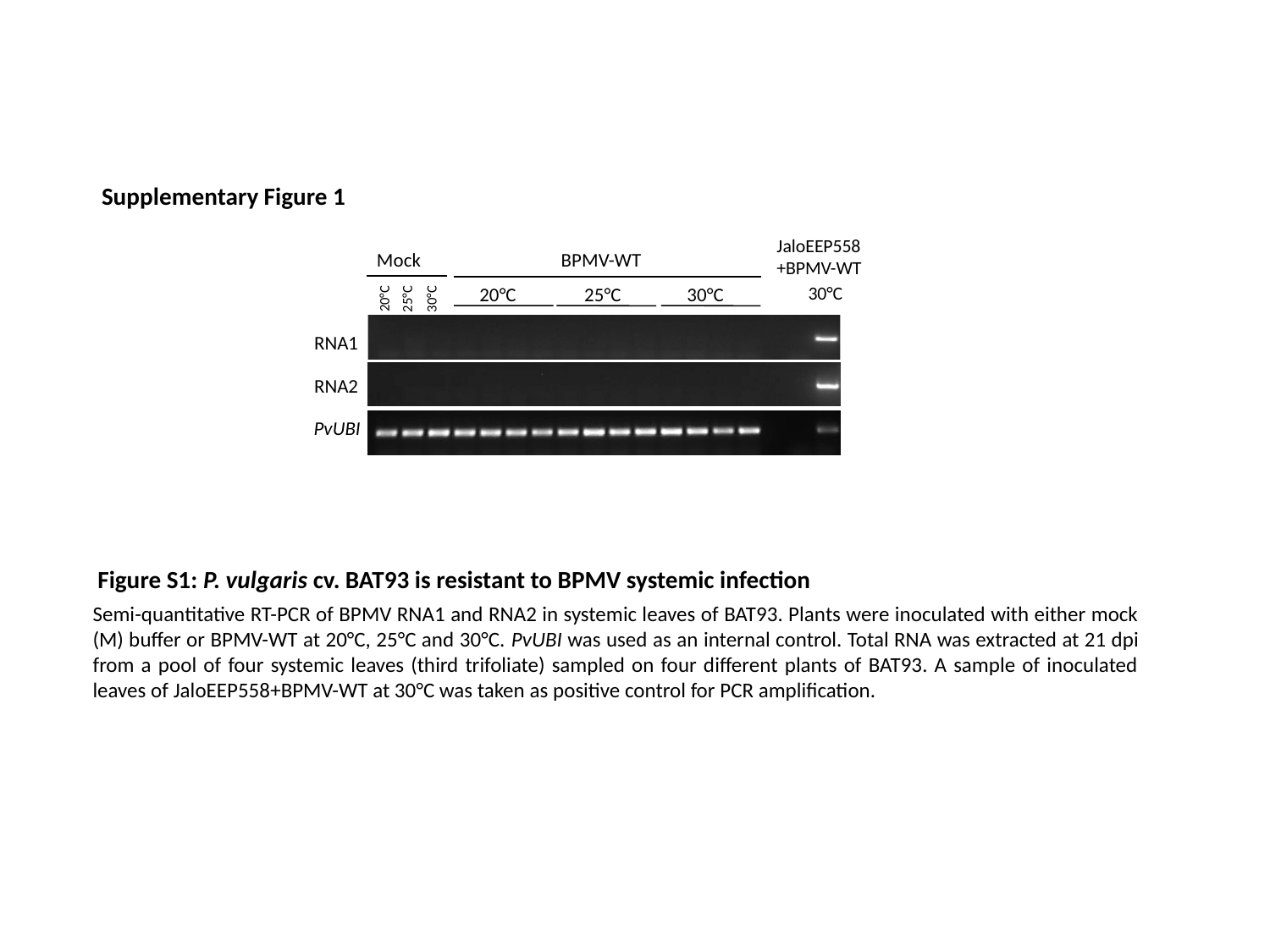

Supplementary Figure 1
JaloEEP558
+BPMV-WT
Mock
BPMV-WT
30°C
20°C
25°C
30°C
20°C
30°C
25°C
RNA1
RNA2
PvUBI
Figure S1: P. vulgaris cv. BAT93 is resistant to BPMV systemic infection
Semi-quantitative RT-PCR of BPMV RNA1 and RNA2 in systemic leaves of BAT93. Plants were inoculated with either mock (M) buffer or BPMV-WT at 20°C, 25°C and 30°C. PvUBI was used as an internal control. Total RNA was extracted at 21 dpi from a pool of four systemic leaves (third trifoliate) sampled on four different plants of BAT93. A sample of inoculated leaves of JaloEEP558+BPMV-WT at 30°C was taken as positive control for PCR amplification.

## Slide 2
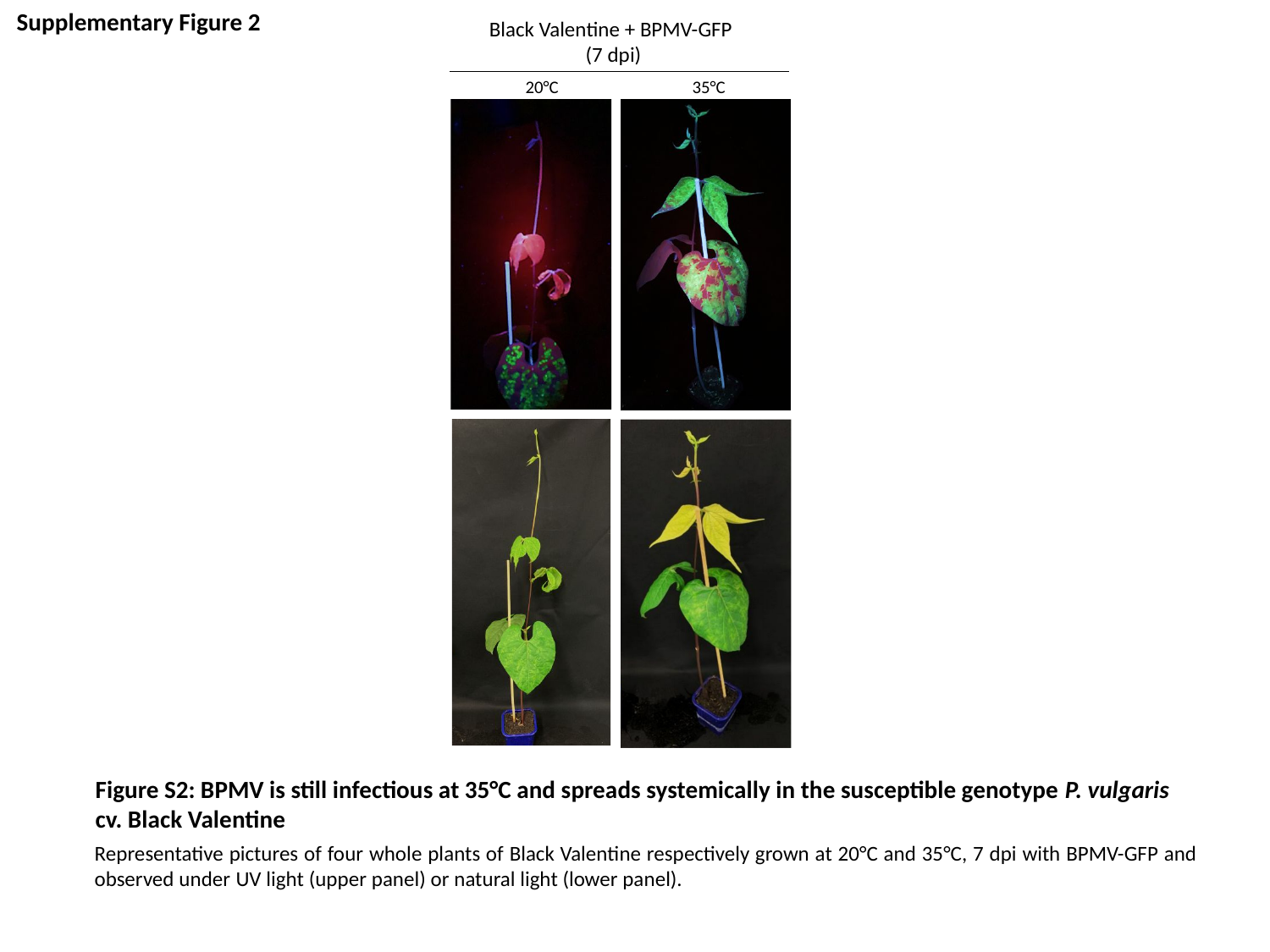

Supplementary Figure 2
Black Valentine + BPMV-GFP
(7 dpi)
 20°C	 35°C
Figure S2: BPMV is still infectious at 35°C and spreads systemically in the susceptible genotype P. vulgaris cv. Black Valentine
Representative pictures of four whole plants of Black Valentine respectively grown at 20°C and 35°C, 7 dpi with BPMV-GFP and observed under UV light (upper panel) or natural light (lower panel).

## Slide 3
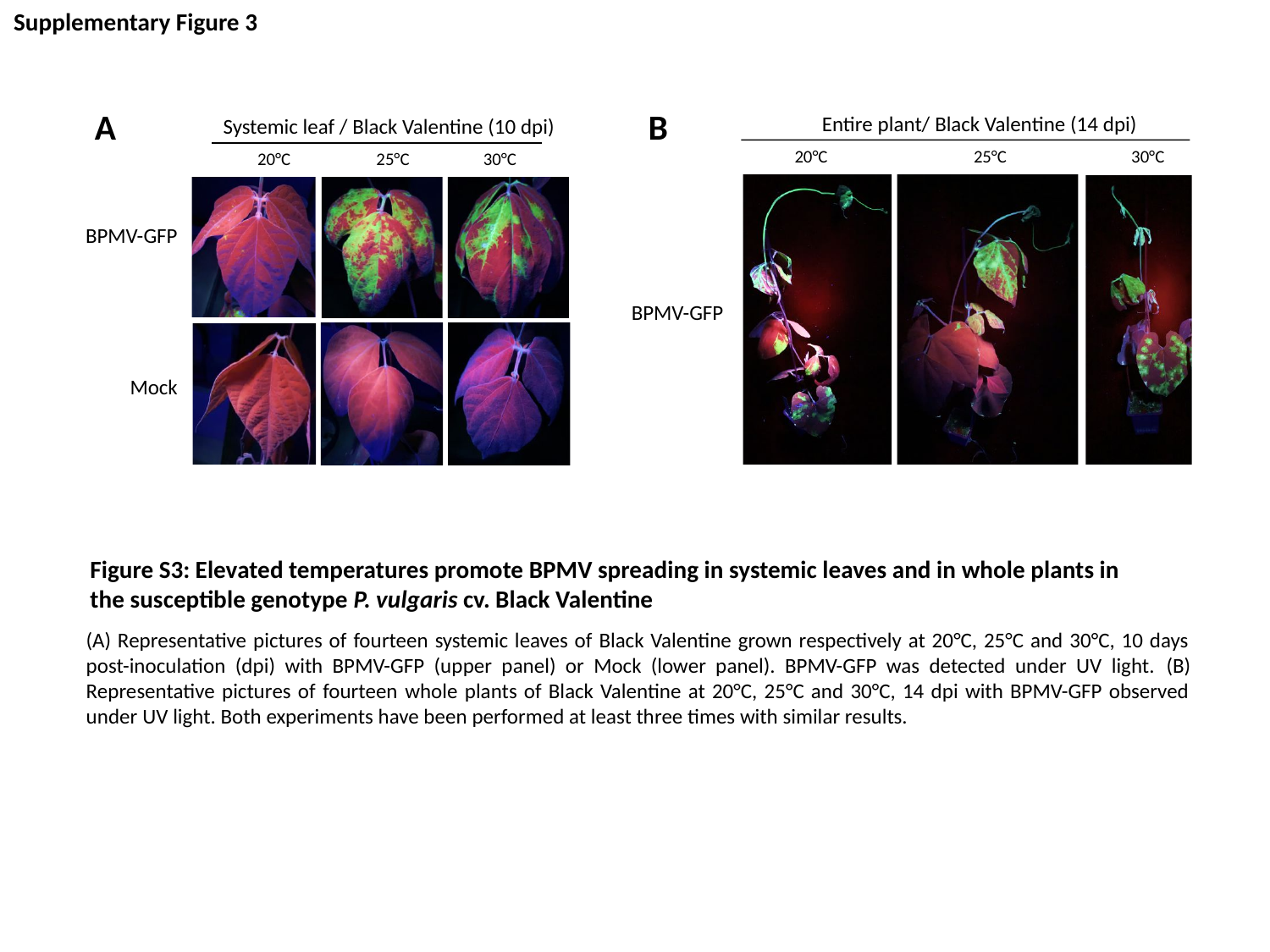

Supplementary Figure 3
A
B
Entire plant/ Black Valentine (14 dpi)
Systemic leaf / Black Valentine (10 dpi)
 20°C	 25°C	 30°C
 20°C	 25°C	 30°C
BPMV-GFP
Mock
BPMV-GFP
Figure S3: Elevated temperatures promote BPMV spreading in systemic leaves and in whole plants in the susceptible genotype P. vulgaris cv. Black Valentine
(A) Representative pictures of fourteen systemic leaves of Black Valentine grown respectively at 20°C, 25°C and 30°C, 10 days post-inoculation (dpi) with BPMV-GFP (upper panel) or Mock (lower panel). BPMV-GFP was detected under UV light. (B) Representative pictures of fourteen whole plants of Black Valentine at 20°C, 25°C and 30°C, 14 dpi with BPMV-GFP observed under UV light. Both experiments have been performed at least three times with similar results.

## Slide 4
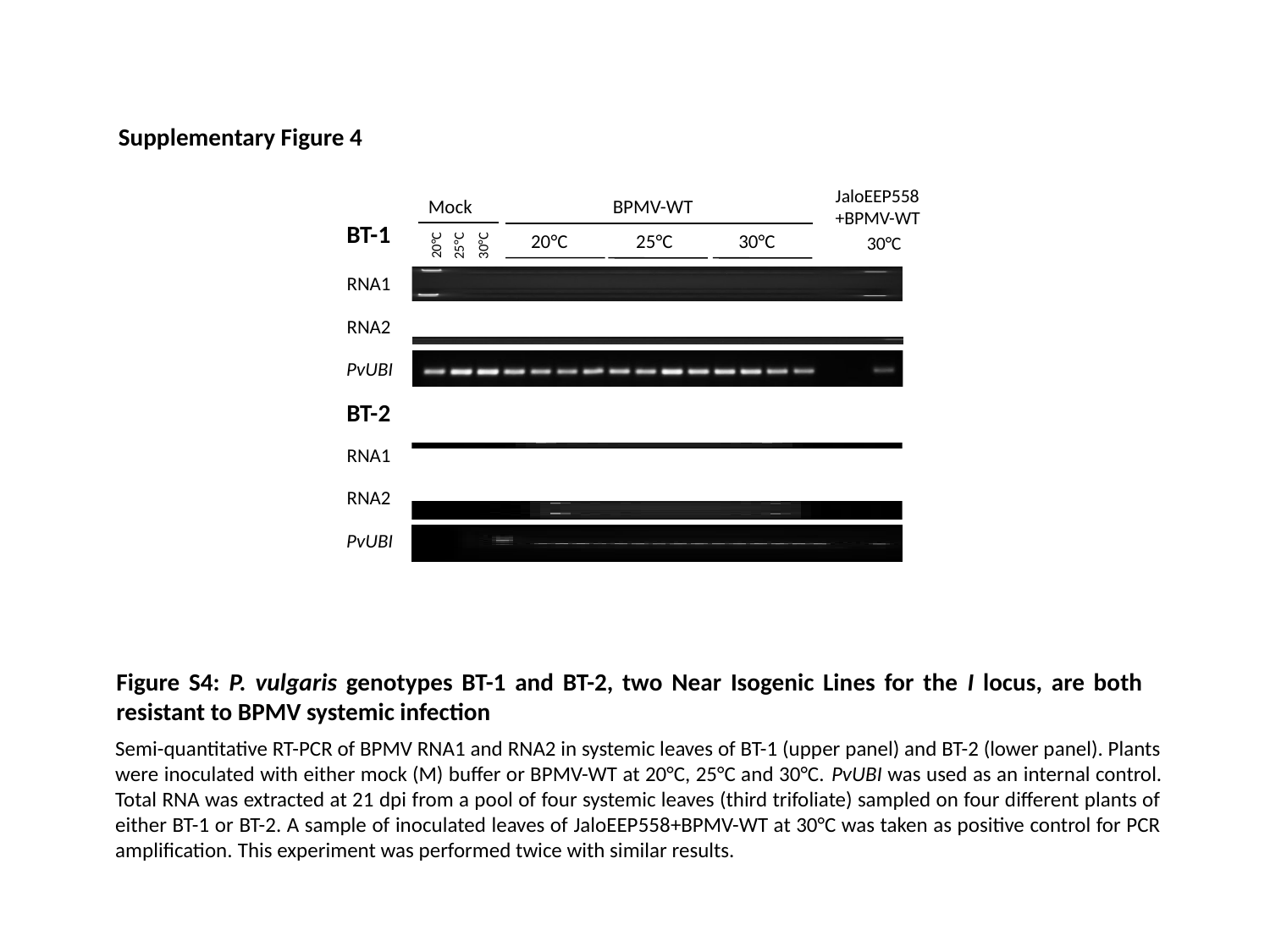

Supplementary Figure 4
JaloEEP558
+BPMV-WT
Mock
BPMV-WT
BT-1
20°C
25°C
30°C
30°C
20°C
30°C
25°C
RNA1
RNA2
PvUBI
BT-2
RNA1
RNA2
PvUBI
Figure S4: P. vulgaris genotypes BT-1 and BT-2, two Near Isogenic Lines for the I locus, are both resistant to BPMV systemic infection
Semi-quantitative RT-PCR of BPMV RNA1 and RNA2 in systemic leaves of BT-1 (upper panel) and BT-2 (lower panel). Plants were inoculated with either mock (M) buffer or BPMV-WT at 20°C, 25°C and 30°C. PvUBI was used as an internal control. Total RNA was extracted at 21 dpi from a pool of four systemic leaves (third trifoliate) sampled on four different plants of either BT-1 or BT-2. A sample of inoculated leaves of JaloEEP558+BPMV-WT at 30°C was taken as positive control for PCR amplification. This experiment was performed twice with similar results.
